# Assessment of Preference and Its Determinant Factors to Ward Modern Contraceptive Methods among Women of Reproductive Age Group in Shire Indaselassie Town, Northern Ethiopia, 2011

**DOI:** 10.1155/2013/317609

**Published:** 2013-12-11

**Authors:** Weyzer T. Tsehaye, Daniel Mengistu, Emebet Birhanu, Kalayou K. Berhe

**Affiliations:** ^1^Department of Nursing, College of Health Sciences, Mekelle University, P.O. Box 1871, Tigray Region, Ethiopia; ^2^Department of Nursing and Midwifery, College of Health Sciences, Addis Ababa University, P.O. Box 1171, Addis Ababa, Ethiopia

## Abstract

*Background*. Women's preferences for various contraceptive methods attribute vary according to the type of relations and other aspects of their life. The discrepancy between fertility preferences and contraceptive practice is regarded as an indicator of unmet demand for family planning. *Objective*. To assess modern contraceptive methods preference and its determinant factors among women of reproductive age group in Shire Indaselassie town, Tigray Region, Northern Ethiopia. *Method*. A community based cross-sectional study design was employed on 367 sampled women. Stratified sampling technique was used to select the study subjects. Then, data was collected using structured questionnaire. *Result*. In this study, the most commonly preferred modern contraceptive method was injectable contraceptive 202 (55%), the second 61 (16.6%) was oral contraceptives, and the third 47 (12.8%) was Norplant. Condom 31 (8.4%), IUD 14 (3.8%), female sterilization 7 (1.9%), and others were less commonly preferred methods. Some of the reasons for preference were effectiveness of the method, reversibility, fewer side effects, convenience, long duration of use, and no need to remember daily. *Conclusion*. This study clearly described that women preferences of modern contraceptive methods increased after they had higher number of children and less desire to limit family size.

## 1. Introduction

### 1.1. Background

Worldwide contraceptive prevalence is estimated to be 58% in 1993. In the more developed countries, regional prevalence variations fall within a relatively narrow range, from 69% in Eastern and Southern Europe to 78% in northern Europe. Among the less developed countries, contraceptive prevalence is the lowest in Africa. Use of contraception among married women in less developed countries varies from a low 8% in Western Africa to a high 83% in Eastern Asia. Modern methods account for the majority of currently global contraceptive practice; almost it covers 9 out of every 10 contraceptive users. Contraceptive prevalence at the global level will need to be at least 66%–75% in the more developed regions and 67% in the less developed regions to attain the projected decline in fertility by the year 2025. Those estimates imply a nearly 60 percent increase in the number of contraceptive users among married women. The largest proportional increase will be in Africa where projections call for the number of users to more than double up to 2005 and to continue to increase rapidly. Ethiopia is one of the Sub-Saharan countries with alarming population growth rate of 2.7% and the total fertility rate is nearly six. Fertility preferences 78% of married women say that they either want to delay the birth of their next child or to have no more children (including those sterilized). Fertility preferences are closely related to the number of living children a woman has. In general, as the number of living children increases, the desire to want another child decreases. For example, 58% of currently married women with 5 living children say they want to have no more children or have been sterilized, in contrast to 9% of women with no children [1–6].

### 1.2. Problem Statement

The highest total fertility rate is observed in Sub-Saharan Africa at 5.4 percent, followed by Latin America at 3.1 percent and Asia at 3.0 percent given such uncontrolled population growth and its impact on the socioeconomic development of the society, great emphasis has been given to family planning, which plays role in reducing fertility worldwide. Inadequate family planning strategies have continuously exacerbated the vulnerability of developing countries, culminating into high maternal and infant mortality. The increase of poverty, disintegration of the extended family system, high incidence of HIV/AIDS and sexually transmitted infections, and high incidence of morbidity and mortality. At least 25% of all maternal deaths can be prevented by family planning. One in four infant deaths in developing countries can be prevented by spacing birth at least two years apart [1, 3, 5].

Ethiopia's reproductive health indicators showed that the total fertility rate is 5.9 children per women. Maternal mortality ratio (MMR) stands among the highest in the world with 850 maternal deaths per 100,000 live births in 1999 and only 6% of the women in the reproductive age use modern contraceptives in 1999 and MMR reduced to 673 per 10,000 live births in 2005. Contraceptive use differs significantly across regions, with about 3% of women in the Somali region reporting using modern contraception compared to about 60% in Addis Ababa. Urban women are five times more likely to use contraceptives than rural women. The most popular modern methods of contraception are implants and the contraceptive pills. Less than 1% of currently married adolescents aged 15–19 and 1% of currently married women aged 20–24 reported using a condom as a family planning method. The unmet needs for 15–19-year-old women are twice as high as the unmet needs for women aged 45–49 [3–5, 7–9].

According to the FMOH health indicators report EFY 2001, the distribution of contraceptives was as follows: pill 3.8%, Depo-Provera 15.5%, Implant 72.7%, IUCD 0.2%, condom 5%, and other methods 2.7%. Contraceptive prevalence of Tigray was 67.8% and the prevalence of Shire Indaselassie town was 48% in 2010. Modern contraceptive coverage in Shire Indaselassie town was 48% in 2002. The percentages of different contraceptive methods used by women in Shire town according to annual performance report of the town's health office was as follows: pill 10%, Depo-Provera 76%, Norplant 6%, IUD 1%, male condom 7%, and permanent (sterilization) methods of contraception are not offered. Although the family planning services are available in most places, the national as well as the regional CPR is still low. In addition, there is insufficient distribution of modern contraception and a wide range of modern contraceptive choices is also lacking to meet the demand of clients. Therefore, the purpose of this study was to assess the preference and its determinant factors to ward modern contraceptive methods among women of reproductive age group in Shire Indaselassie town, northern Ethiopia.

## 2. Method and Materials

### 2.1. Study Area and Period

The study was conducted in the northern part of Ethiopia, Tigray region, Shire Indaselassie town from October 2010 to May 2011. Shire Indaselassie town is situated 1087 km away from Addis Ababa in northern Ethiopia. The town is found in the northern west direction at a distance of 304 km from Mekelle capital city of Tigray regional state. The town has one hospital, one governmental health center, and one MCH center managed by both the regional government and nongovernmental organization. According to 2006/07 national census report, the total population of Shire Indaselassie town was 51,197 of which 26,031 are females. Women of child-bearing age (15–49 years) were 12,031 and households were 11,636.

### 2.2. Source and Study Population, Sample Size, and Eligibility Criteria

The source population was all women of reproductive age group (15–49) who reside in Shire Indaselassie town. Study population was sampled women of reproductive age group from the selected three kebeles in the town. The sample size was calculated by using the single population proportion formula and by considering Tigray region contraceptive coverage of 68% with 95% confidence interval at marginal error of 5% and 10% of nonrespondents, and then the final sample size was 367. Inclusive criteria were all women whose ages were within reproductive age group (15–49) and who was willing to participate in the study and can give consent. And all women who were pregnant, critically ill, and involuntary to participate were excluded from the study.

### 2.3. Sampling Procedures and Techniques

Stratified sampling technique was used to select the study participants. From 5 kebeles, three kebeles (kebeles 01, 03, and 05) were randomly selected using lottery method. Then, the study subjects' household was selected through systematic sampling technique at every “*k*ith” interval, whereas the first house hold was selected by lottery method, then continuing to every *K*th house hold; if there was no respondent in the house hold, we continue to the next house until the desired sample size was attained.

### 2.4. Data Collection Tools and Procedures

Structured Interviewer administer was modified by the principal investigator from a previous study done in Bahir Dar and the questionnaire consists of three parts. The first part contained socioeconomic and demographic questions, the second part contained reproductive history, and the last consists of questions related to contraceptive interaction. Fifteen voluntary community health workers and high school students were assigned to collect the required data through face to face interview. Data collectors got the willingness of participants after explaining the purpose of conducting this research and consent was granted from the participants before data collection.

### 2.5. Variables of the Study and Operational Definitions

Dependent variables were modern contraceptive practice or utilization and independent variables were sociodemographic characteristics like age, religion, ethnicity, marital status, literacy status, occupation, monthly income, and travel distance to health care center. Reproductive history like parity, gravidity, birth interval, still birth, abortion, age at first pregnancy, age when you get married, number of live children, client method of contraceptive interaction like ease and convenient time of using contraceptive, convenience of the routine activity, discontinuing using contraceptive, and self-assessment after using contraceptive. Operational definitions were as follows: utilization: use of any modern contraceptive method to space the child and to protect unwanted pregnancy.

### 2.6. Data Quality Assurance

The structured questionnaire was translated to Tigrigna and back to English to maintain its consistency. Pretest was undertaken in Adidaero town, which was not included in this study. Based on the pretest findings, the instrument was revised, edited, and modified according to the result of the pretest. Data collectors and supervisors were trained for one day on the objective of the study, contents, consistence, and logical order of the questionnaire and how to maintain confidentiality and privacy of the participants. The collected data was checked by supervisors and principal investigator on a daily basis. In the case of the concurrence of incompleteness and inconsistence, data collectors went back to respondent's house to refill the questionnaire.

### 2.7. Data Processing and Analysis

After data collection was completed, data entry, cleaning, and analysis were done by using SPSS version 16 program in order to describe the descriptive statistic in relation to variables and frequencies, mean, standard deviation, and percentages were computed. To determine the association between the dependent and independent variables using statistical analysis, binary and multivariant logistic regression were computed, and, in this statistical analysis, association was determined, if *P* value was less than 0.05 and it was considered as statically significant.

### 2.8. Ethical Consideration

Approval of ethical clearance was granted from Addis Ababa University, College of Health Sciences, Department of Nursing and Midwifery Ethical Review Committee. Letter of permission was also obtained from Tigray Regional State Health Bureau and Shire Indaselassie town health office. Written consent was also received from study subject after being provided adequate information by reading the information sheet that describes the purpose of the study and its confidentiality and beneficence. In order to ensure the confidentiality of the information, all data were kept in secret and coded in anonymity.

## 3. Result

### 3.1. Sociodemographic Characteristics

In this study 367 reproductive age group women participated. All distributed questionnaires were filled completely and consistently. This made the response rate 100%. Out of the total study subjects 106 (28.9%) were aged 25–29 years, whereas 17 (4.6%) were found to be aged 45–49 years. Nearly 87% were Tigrian. Regarding religion 278 (75.7%) respondents were orthodox. Regarding educational level 79 (21.5%) respondents were above high school. The majority, 229 (62.4%), was married. One hundred thirty-four (36.5%) were private workers, while 86 (23.4%) were merchants. More than half 214 (58.3%) monthly income were 100–500 Birr whereas 82 (22.3%) were 501–1000 Birr. 152 (41.4%) participants were residing 11–20 minutes walking distance from the family planning service. Nearly 65% of study subjects resided 5–10 minutes distance by car from family planning while 152 (41.4%) were 11–20 minutes (See Table 1).

### 3.2. Reproduction Related Variables

Reproduction and fertility related variables of women of reproductive age groups were considered. Among the study subjects, most of them got married, 216 (70.8%), between the age 15 and 19 and only 1 (0.3%) got married at 35 and above. Most of respondents, 314 (85.6%), reported that they were pregnant in their lifetime. Of this majority, 280 (89.2%) reported that they were pregnant after marriage and 27 (79.4%) had had 1-2 times of pregnancy before marriage. Majority of them, 190 (60.5%), had got pregnant for the first time and were found to be between 15 and 19 years of age while only one respondent had got her first pregnancy between age groups of 35 and above. Regarding age of women when their first child was born, 143 (45.5%) were found to be between 20 and 24 years and the least 2 (0.6%) were found in age groups of 35 and above. 183 (58.3%) mothers have had 1-2 numbers of live births and the least 11 (3.5%) have had 8 and above numbers of live births. Among the respondents 61 (16.6%) and 85 (23.2%) experienced still births and abortions in their lifetime, respectively. The greatest average birth interval was between 24 and 35 months of age while the least was 72 months (6 years). More than half of the respondents, 210 (57.2%), wanted to have 3–5 numbers of children and 42 respondents (11.4%) perceived having 1-2 numbers of children (see Table 2).

### 3.3. Preference and Source of Modern Contraceptives

In this study, the most commonly preferred modern contraceptive method was injectable 202 (55%), the second 61 (16.6%) was oral contraceptives, and the third 47 (12.8%) was Norplant. Condom 31 (8.4%), IUD 14 (3.8%), female sterilization 7 (1.9%), and others were less commonly preferred methods. Two hundred sixty-nine (91.5%) of respondents had got contraceptive method according to their choice and only 25 (8.5%) had got without their choice. The major sources of supply of MCM 95 (32.7%) were from governmental health center and the second most important source of supply was hospital followed by MCH health center (NGO), 46 (15.3%), and health extension workers, 48 (15.3%). The majority of respondents preferred governmental institution (health center 127 (34.6%), health extension 105 (28.6%), and hospital 70 (19.1%) followed by FGAE 21 (5.7%) and NGO 21 (5.7%) and others (private clinic and pharmacy) composed least proportion (see Table 3)).

### 3.4. Reason for Preferring Pill

The most commonly mentioned reason for preferring pill among women in the reproductive age group from the given options is as follows. 42 respondents (64.6%) said the used it because of the effectiveness of pill followed by 40 (61.5%) who said it is reversible, and 38 (58.5%) reported that it has fewer side effects and 29 (44.6%) said it is convenient. Regarding daily dose of pills, most respondents 57 (87.7%) answered one time per day, and 6 (9.2%) answered that they did not know how many times they should take pills and 2 (3.1%) did not give responses. The respondents were also asked, what they would do if they forgot daily dose of pills; the majority of respondents 34 (52.3%) answered correctly that they take pills as soon as they remembered and take it together with the next dose at regular time while least of them 1 (1.5%) did not give responses (Table 4).

### 3.5. Reason for Preferring IUD

Many reasons were given for preferring IUD during interview. The majority of women mentioned more than one reason. The most common reason mentioned was as they, 14 (100%), did not need replacing IUD before 8 years and 8 respondents (57.1%) reported IUD was preferred due to its reversibility; half of them 7 (50%) preferred that it had no effect during lactation and 5 respondents (35.7%) reported nothing to remember constantly; 2 (14.3%) reported that it was very effective and 2 respondents (14.3%) mentioned other reasons. The main reason that respondents 14 (100%) came to health institution before their appointment was due to heavy bleeding and irregular menstrual cycle and 13 respondents (92.9%) due to pelvic or abdominal pain followed by fever within 2-3 days and only respondent (7.1%) did not know the reason for preferring IUD.

### 3.6. Reason for Preferring Injectable

Choices were given for the reason of preferring injectable. Most women choose more than one reason. The highest choice of respondents 135 (66.8%) was no need to daily remember other contraceptives. The second reason 33 (16.3%) was its high effectiveness 122 (60.4%) and the least 33 respondents (16.3%) were due to other reasons. Majority of respondents 184 (91.1%) and 17 (8.4%) answered that it was taken every three and two months, respectively, and one respondent answered that injectable was taken at any time. Several respondents 158 (78.2%) who came back to health institution out of normal program were due to amenorrhea, 154 (76.2%) prolonged or heavy bleeding, 111 (55%) headache (persistent) and the least 22 (10.9%) (See Table 5).

### 3.7. Reason for Preferring Norplant

Among the study subjects, 40 respondents (85.1%) reported that they preferred it due to no need of remembrance and 37 (78.7%) preferred that it was long acting, and few of them 6 (12.8%) preferred it due to other reasons. Out of 47 (12.8%) respondents preferring Norplant, 19 respondents (40.4%) answered that it was left inside for three years after insertion and 17 respondent (36.2%) answered for five years and 11 (23.4%) answered that they did not know for how long it stayed. In addition, 42 respondents (89.4%) mentioned that the reasons for which they would go to health institution were irregular and heavy bleeding and 35 (74.5%) mentioned headache (severe) and 2 (4.3%) had no response.

### 3.8. Reason for Preferring Condom, Female Sterilization, and Spermicidal

The reasons for preferring condom method among the respondents were different. But most women 30 (96.8%) responded that condom was preferred as it protects against STI, 29 (93.5%) responded that it protects against HIV/AIDS, 15 (48.4%) preferred it due to its accessibility, and the rest 11 (35.5%) preferred that it was convenient. On the other hand, majority of the study subjects 7 (100%) decided not to have more children. So, all seven women preferred female sterilization method of contraceptive. From the study subjects the highest response was the effectiveness of spermicidal, if it was used together with condom 3 (100%) and 2 respondents (66.7%) preferred spermicidal due to its fewer side effects.

### 3.9. Association between Modern Contraceptive Method for Preference of (Pill, Injectable, and Norplant)

Regarding this study subject age 20–24 years and 30–34 years, were 0.12–0.27 times more likely to preferred pills as compared to 15–19 years with ADR = 0.12, 95% CI (0.032, 456), 0.27 95% (0.85, 880), respectively. Married women were 5.3 times more likely to prefer injectable ADR = 5.3 95% CI (2.360, 12.140), than single. And also married and widowed women were 0.17-0.13 times more likely to prefer Norplant, ADR = 0.17, 95% CI (067, 470), 0.13 (038, 473), than single women (see Table 6).

## 4. Discussion

One of the eight millennium developmental goal is improving maternal health through reducing maternal mortality by two-third. This could be achieved by quality reproductive health and family planning service. Family planning improves community health by helping both men and women to have children when they are physically, emotionally, and financially prepared to take the child bring up responsibility. As IPPF gave great attention to reproductive health for best quality service improvement accessibility, acceptability, and convenience are important for contraceptive users. This study focused on assessment of preference and its determinant factors to ward modern contraceptive methods among women of reproductive age group. The study area Shire Indaselassie town has showed significant difference in sociodemographic characteristics such as age, marital status, and education. There was also a statistically significant difference by some reproductive characteristics such as age at time of marriage, number of live births, total number of children, and number of children you want to have. The age of respondents was less likely associated with the preference of pills. This is similar to the study conducted in Bahrdar town in 2005, that the age of respondents was negatively associated [4].

Regarding marital status, the result showed that most respondents were married (62.4%) and were 2.70 times more likely to use modern contraceptive than unmarried women. This is unlike the study done in South Wollo zone, in 2010, which was 82.9% among married women [10, 11]. Even in the preference of contraceptive, married women were 5.35 times more likely to prefer injectable as compared with unmarried women, but it showed that married women were less likely to prefer Norplant contraceptives than single women. This is unlike the study result in south east Ethiopia, in 2004, which revealed that married women were more likely to prefer Norplant than single women. This might be related to the probability that the married women need to conceive child within short period of time, since injectable has shorter duration than Norplant [12].

In study area the majority of women preferred MCM (61%) injectable and the least (0.3) male sterilization. This shows they were aware of family planning and they have knowledge about the different methods. Like a study done in Woreta town, Ethiopia, in 2008, the most commonly preferred MCM (63.2%) was injectable [13]. Unlike a study done in Turkey 70% women were using family planning method where as 30% were not using. The most preferred method was coitus interruptus. In addition the study show in Iran in all 300 women who were using withdrawal took part in the study of these, 210 women (70%). The most common reasons for using withdrawal were having no cost involvement, having no need for medical advice, having fewer side effects, and being easier to use than other methods [14, 15].

## 5. Conclusion and Recommendation

### 5.1. Conclusion

Injectable was the most commonly preferred method followed by oral contraceptive pill and Norplant in the study areas. Being married was positively associated with the preference of injectable whereas ages 20–24 and 30–34 were negatively associated with the preference of pills. Being married and widowed was negatively associated with preference of Norplant modern contraceptive. Clients 269 (91%) were highly likely to get the method of their choice in study area; only 25 (8.5%) less get according their preference.

### 5.2. Recommendation

#### 5.2.1. To Regional Health Bureau and MOH

Based on the evidence (findings) obtained from the present study the following specific interventions are recommended. Not only should family planning intervention consider the health aspect but also demographic and reproductive rights should be equally considered and collaboration of relevant bodies to a very high fertility desire of population is mandatory. Intervention strategies that aimed to control reproductive and fertility should be according to the magnitude of the problem in different ways for the population. Information, education, and communication (IEC) activities regarding family planning service should be strengthened by the MOH and RH through mass media messages, encouraging and broadening the activities of health workers in the study area. It is recommended to include MCMs in the educational curriculum both at elementary and secondary schools by education bureaus so that early knowledge and practice of MCU can materialize at least for those who are not out of school. Sustainable resources especially injectable contraceptive should be ensured.

#### 5.2.2. To Woreda Health Office

Family Planning IEC programs in Woreda should target women before marriage in every possible way in schools at junior level and above, because women in Woreda go for child spacing and birth limiting after they had all the pregnancies and number of children they wanted. Since the utilization is good the Woreda health office should maintain and encourage the pattern.

## Figures and Tables

**Table 1:** Distribution of study subjects by socioeconomic and demographic characteristics among reproductive age group in Shire Endaslasie town (*n* = 367), northern Ethiopia, 2011.

Variables	Frequency	Percent
Age in years		
15–19	23	6.3
20–24	66	18.0
25–29	106	28.9
30–34	55	15.0
35–39	61	16.6
40–44	39	10.6
45–49	17	4.6
Ethnicity		
Tigray	318	86.6
Amhara	32	8.7
Oromia	9	2.5
Eretria	8	2.2
Religion		
Orthodox	278	75.7
Muslim	69	18.8
Catholic	24	3.8
Protestant	6	1.6
Marital status		
Married	229	62.4
Single	62	16.9
Widowed	44	12.0
Divorced	32	8.7
Educational status		
Illiterate	75	20.4
Read and write	46	12.5
Elementary	101	27.5
High school	66	18.0
Above high school	79	21.5
Occupation		
Government employees	54	14.7
Retired	19	5.2
Private worker	134	36.5
Merchant	86	23.4
Farmer	7	1.9
Housewife	67	18.3

**Table 2:** Distribution of study subjects' reproductive history among reproductive age group in Shire Endaslasie town, northern Ethiopia, 2011.

Variables	Frequency	Percent
Age when she get married (*n* = 367)		
Less than 15 years	41	13.4
15–19 years	216	70.8
20–24 years	37	12.1
25–29 years	10	3.3
30–34 years	62	16.9
And above 35 years	1	0.3
Had pregnancy (*n* = 367)		
Yes	314	85.6
No	53	14.4
Pregnancy in relation to marriage (*n* = 314)		
Before marriage	34	10.8
After marriage	280	89.2
Number of pregnancies before marriage (*n* = 34)		
1-2 times	27	79.4
3-4 times	6	17.6
5 and above	1	2.9
Age in the first pregnancy (*n* = 314)		
Less than 15 years	12	3.8
15–19 years	190	60.5
20–24 years	89	28.3
25–29 years	20	6.4
30–34 years	2	0.6
And above 35 years	1	0.3
The age your first child was born (*n* = 314)		
Less than 15 years	9	2.9
15–19 years	124	39.5
20–24 years	143	45.5
25–29 years	33	10.5
30–34 years	3	1.0
And above 35 years	2	0.6
Number of live births (*n* = 314)		
1-2 children	183	58.3
3–5 children	94	29.9
6-7 children	26	8.3
≥8 children	11	3.5
The number of live children in total (*n* = 314)		
1-2 children	169	53.8
3–5 children	111	35.4
6-7 children	25	8.0
8 and above children	9	2.9
Birth interval (*n* = 314)		
<12 months	38	12.1
12–23 months	94	29.9
24–35 months	120	38.2
36–47 months	22	7.0
48–59 months	15	4.8
60–71 months	13	4.1
72 and above	12	3.8
The number of children you want to have (*n* = 367)		
1-2 children	42	11.4
3–5 children	210	57.2
6–8 children	115	31.4

**Table 3:** Distribution of study subjects on preference of modern contraceptives related variables among women of reproductive age group in Shire Endaslasie town, northern Ethiopia, 2011.

Variables	Frequency	Percent
Getting modern contraceptive methods of your choice (for current users) (*n* = 294)		
Yes	269	91.5
No	25	8.5
The place that you get modern contraceptive method (for users) (*n* = 294)		
From private clinic	11	4.6
From government hospital	64	22.0
From health extension	48	14.7
From health center	95	32.7
From FGAE	6	2.1
From NGO	46	15.3
From pharmacy/drug vendor	24	8.6
The place that you prefer to get family planning service (*n* = 367)		
From private clinic	17	4.6
From government hospital	70	19.1
From health center	127	34.6
From health extension	105	28.6
From FGAE	21	5.7
From NGO	21	5.7
From pharmacy/drug vendor	6	1.6

**Table 4:** Distribution of reasons for pill preference among reproductive age group in Shire Endaslasie town (*n* = 65), northern Ethiopia, in March, 2011.

Variables	Frequency	Percent
Very effective		
Yes	42	64.6
No	23	35.4
It is convenient		
Yes	29	44.6
No	36	55.4
Reversible		
Yes	40	61.5
No	25	38.5
Fewer side effects		
Yes	38	58.5
No	27	41.5
Easily available		
Yes	25	38.5
No	40	61.5
Does not affect lactation		
Yes	12	18.5
No	53	81.5
The time of taking pill		
Do not know	57	87.7
One every day	6	9.2
No response	2	3.1
The action for missed pills for one day		
Take it as soon as you remembered it/take it together with the next dose at regular time	34	52.3
Take only the next dose at regular time	10	15.4
Discontinue to take the rest of the pills	4	6.2
Do not know	16	24.6
No response	1	1.5

**Table 5:** Distribution of reasons why injectable contraceptive methods are preferred among reproductive age group in Shire Endaslasie town (*n* = 202), northern Ethiopia, 2011.

Variables	Frequency	Percent
How often do you get injectable contraceptive?		
Every two months	17	8.4
Every three months	184	91.1
Any other time	1	0.5
When which problem appears do you come back to health institution?		
Headache (persistent)		
Yes	111	55.0
No	91	45.0
Weight gain		
Yes	90	44.6
No	112	55.4
Amenorrhea		
Yes	158	78.2
No	44	21.8
Prolonged or heavy bleeding		
Yes	154	76.2
No	48	23.8
Do not know		
Yes	47	23.3
No	155	76.7
No response		
Yes	22	10.9
No	180	89.1

**Table 6:** Association between modern contraceptive method for preference of (pill, injectable and Norplant) among reproductive age group women, Shire Endaslasie town, northern Ethiopia, 2011.

	COR (95% CI), *P*-value	Adjusted (95% CI), *P*-value
Pill as dependent Yes = 61 (16.6%) No = 306 (83.4%)		
Age in years*		
15–19	1.00	1.00
**20–24**	0.093, (0.022, 0.399), 0.001	**0.121, (0.032, 0.456), **0.002*
**25–29**	0.286, (0.091, 0.904), 0.033	0.409, (0.152, 1.103), 0.077
**30–34**	0.186, (0.047, 0.736), 0.017	**0.273, (0.085, 0.880), **0.030*
35–39	0.315, (0.084, 1.175), 0.085	0.413, (0.140, 1.212), 0.107
40–44	0.431, (0.115, 1.620), 0.213	0.563, (0.181, 1.752), 0.321
45–49	0.292, (0.052, 1.645), 0.163	0.402, (0.088, 1.825), 0.238
Injectable as dependent Yes = 202 (55%) No = 16.5 (45%)		
Marital status*		
Single	1.00	1.00
**Married**	0.317, (0.166, 0.608), 0.001	**5.35, (2.360, 12.140), **0.000*
Widowed	0.279, (0.129, 0.604), 0.001	1.61, (0.640, 4.069), 0.310
Divorced	0.168,( 0.069, 0.411), 0.000	1.19, (0.440, 3.236), 0.729
Norplant as dependent Yes = 47 (12.8%) No = 320 (87.2%)		
Marital status*		
Single	1.00	1.00
**Married**	0.590, (0.191, 1.827), 0.361	**0.177, (0.067, 0.470), **0.001*
**Widowed**	2.055, (0.717, 5.892), 0.180	**0.134, (0.038, 0.473), **0.002*
Divorced	5.386, (1.774, 16.356), 0.003	0.454, (0.143, 1.444), 0.181
Occupation*		
Government employees	1.00	1.00
Retired	2.522,(0.437, 14.557), 0.301	1.065, (0.397, 2.857), 0.901
Private worker	1.043, (0.310, 3.514), 0.946	1.171, (0.318, 4.317), 0.813
Merchant	0.236, (0.053, 1.049), 0.058	0.691, (0.299, 1.595), 0.386
Farmer	0.000, (0.000, —), 0.999	0.169, (0.048, 0.593), 0.006
House wife	1.044, (0.265, 4.114), 0.951	0.000, (0.000, —), 0.999

N.B: *showed that statistically significant at *P* ≤ 0.05.
